# Iron-respiring bacteria mediate regulatory mechanisms and pathways of electron donors and acceptors in carbon and nitrogen cycles

**DOI:** 10.1128/aem.01007-26

**Published:** 2026-07-22

**Authors:** Yue Dong, Ziyi Gu, Mingrui Sui, Xin Wang, Mingchao Zhu, Yiying Jiang, Jialiang Shi, Peng Zhang, Teta Huguette Dion

**Affiliations:** 1 Key Laboratory of Integrated Regulation and Resource Development on Shallow Lakes of Ministry of Education, College of Environment, Hohai University 626722, Nanjing, China; 2 College of Water Conservancy and Hydropower Engineering, Hohai University 626711, Nanjing, China; 3 MOE Key Laboratory of Pollution Processes and Environmental Criteria and Tianjin Key Laboratory of Environmental Remediation and Pollution Control, College of Environmental Science and Engineering, Nankai University 534792, Tianjin, China; 4 Key Laboratory of Water Safety and Aquatic Ecosystem Health of Xizang Autonomous Region, Xizang Minzu University 377051, Xianyang, China; 5 Water Affairs Department, Guangzhou Baiyun International Airport Company Limited, Guangzhou, China; 6 Tianjin Municipal Ecology and Environment Protection Comprehensive Administrative Law Enforcement Corps, Tianjin, China; 7 Yunnan Key Lab of Soil Carbon Sequestration and Pollution Control, Environmental Science & Engineering, Faculty of Environmental Science & Engineering, Kunming University of Science & Technology, Kunming, China; Colorado School of Mines, Golden, Colorado, USA

**Keywords:** Sediment management, iron-respiring bacteria, carbon and nitrogen cycles, microbial community, metabolic pathways

## Abstract

Iron-respiring bacteria play important roles in global material cycles owing to their electron transfer capabilities, yet their electron-transfer connections to carbon and nitrogen cycles in complex environments remain unclear. Through the investigation of sediments, this study found multidimensional associations between the abundance of iron-respiring bacteria and microbial community structure, as well as carbon and nitrogen cycles. At the community level, the abundance of iron-respiring bacteria showed negative correlations with certain populations, implying a potential influence on population expansion through competition for electron donors. Meanwhile, iron-respiring bacteria might have a symbiotic role that promoted the Shannon index. In terms of cycles, iron-respiring bacteria and carbon metabolism mutually promoted each other, with the *mdh* gene contributing 4.2% to these bacteria. In the nitrogen cycle, the iron transport *sit* gene was widely present in areas with high ammonia nitrogen concentrations. This spatial coupling suggests that it may provide necessary conditions for iron reduction-coupled anaerobic ammonium oxidation (Feammox). Network analysis revealed that iron-respiring bacteria serve as core nodes coupling carbon and nitrogen cycling functional modules. Through potential pathways such as electron-shuttle mediation and their own multifunctional metabolism, they may promote the synergy of carbon and nitrogen reactions. The abundance of iron-respiring bacteria was significantly positively correlated with the electron exchange capacity (EEC) of sediments (*R*² = 0.797), suggesting their possible involvement in forming long-distance electron transfer networks. This study broadens the understanding of potential coupling mechanisms between iron-respiring bacteria and carbon-nitrogen cycling under varying environmental factors and offers new insights into the management of sediments.

**IMPORTANCE:**

Iron‑respiring bacteria in sediments have the potential to drive carbon and nitrogen cycles through electron transfer. They link these cycles by using organic carbon or ammonium as electron donors and iron or nitrate as electron acceptors. Moreover, these bacteria may also produce mobile electron shuttles and have the potential to build long‑distance electron transfer networks within sediments. Electron exchange benefits the metabolism of surrounding microorganisms, helping them decompose recalcitrant contaminants. By understanding the direction of electron flow, we can learn whether the bacteria convert nitrogen into harmless gases or retain it as ammonium, and whether carbon is released as carbon dioxide or stored as carbohydrates. Understanding this electron‑driven coupling provides a scientific basis for the management of lake sediments.

## INTRODUCTION

Sediments, formed by the deposition and adsorption of pollutants, are rich in nitrogen, phosphorus, organic matter, and other substances. They constitute a significant factor influencing lake water quality. Once formed, sediments gradually transition from pollutant sinks to pollutant sources, readily releasing contaminants when disturbed by environmental factors, and are closely linked to carbon and nitrogen cycles (1). Specifically, steps within the nitrogen cycle, such as anammox and denitrification, can convert nitrogen into N₂. Concurrently, organic nitrogen in sediments releases NH₄^+^ via ammonification, exacerbating eutrophication (2). Sediments also serve as the primary site for organic matter mineralization in lakes, where microbial activity releases CO₂ and CH₄ through degassing, establishing them as internal pollution sources (3). Therefore, sustained regulation of these cycles is fundamental to maintaining the long-term effectiveness of lake remediation.

The pathways of the carbon and nitrogen cycles in lake sediments are dynamic and subject to the influence of various environmental factors. Essentially, these cyclic pathways are reactions initiated by microbial electron transfer for metabolism. The electron donors and acceptors present in the environment control the reaction pathways to a certain extent. For instance, studies have demonstrated that the survival of *Methanobacterium* is threatened in the absence of the electron acceptor CO₂, which limits the methanogenesis pathway within the carbon cycle (4). Notably, the metabolism of some microorganisms does not strictly depend on specific electron donors or acceptors. They alter their selection of electron donors and acceptors under different environmental conditions, thereby influencing other cyclic pathways. Specifically, cable bacteria, which metabolize sulfur, tend to utilize O₂ as their terminal electron acceptor. However, when in an anoxic environment, they switch strategies and adopt NO₂^−^ and NO₃^−^ as alternative electron acceptors (5). This process ensures their own sulfur metabolism but subsequently impacts the nitrogen cycle within the sediment and the survival status of other microorganisms. Due to the geographical characteristics of sediments, oxic and anoxic zones coexist, resulting in a diversity of electron donor and acceptor types (6). Consequently, the selection of electron donors or acceptors by microorganisms at various nodes of these cycles is particularly critical. Therefore, from the perspective of electron donors and acceptors, carbon and nitrogen cycles are deeply coupled with the lake environment and other elemental cycles, forming an inseparable whole. However, the dynamics of carbon and nitrogen cycling within this highly coupled system remain to be thoroughly elucidated.

Iron-respiring bacteria, as microorganisms capable of electron transfer, are of great significance to the carbon and nitrogen cycles in sediments. Iron-respiring bacteria primarily consist of two categories: iron-oxidizing bacteria and iron-reducing bacteria, which accomplish their metabolism through the conversion between Fe²^+^ and Fe³^+^. Current research indicates that iron-respiring bacteria possess multiple mechanisms for electron transfer, including the use of electron shuttles, pili, and coenzyme factors, demonstrating potential for ecological remediation (7). Iron-reducing bacteria utilize Fe³^+^ as an electron acceptor and can select various electron donors such as organic matter and sulfides (8). Studies have found that they can participate in the nitrogen cycle through the iron reduction-coupled anaerobic ammonium oxidation (Feammox), a reaction widely present in sediments that oxidizes NH₄^+^ to NO₃^−^ or N₂ (9). Similarly, iron-oxidizing bacteria have been observed to utilize electron transfer in denitrification processes. They select O₂ as an electron acceptor under aerobic conditions but can also utilize NO₃^−^ to complete their metabolism (10). Furthermore, due to the robust extracellular electron transfer capabilities of iron-respiring bacteria, their metabolic processes can indirectly impact the environment. Past research has demonstrated that chelating agents produced during the metabolism of iron-respiring bacteria can accelerate the degradation of aromatic hydrocarbon compounds, indirectly driving the carbon cycle (11). Although the metabolic pathways of iron-respiring bact eria have been extensively studied, the specific mechanisms by which they influence carbon and nitrogen cycles through electron transfer pathways in natural environments remain unclear. Elucidating these mechanisms is crucial for sediment remediation.

This study focused on Dianchi Lake in Yunnan Province, where sediment samples were collected from uniformly distributed sampling points. These samples were subjected to analyses of physicochemical properties and microbial genomics to investigate the coupling relationships among environmental factors, iron-respiring bacteria, and carbon and nitrogen cycling pathways. This study aims to explore (i) the association between iron-respiring bacteria and carbon-nitrogen cycles; (ii) changes in the association between iron-respiring bacteria and carbon-nitrogen cycles under the impact of various environmental factors; and (iii) the mechanisms by which iron-respiring bacteria mediate carbon and nitrogen cycles from the perspective of electron donors and acceptors. This study provides novel insights into carbon and nitrogen cycles in sediments and broadens the understanding of the role of iron-respiring bacteria in these cycles.

## MATERIALS AND METHODS

### Study area and sampling

**Fig 1:**
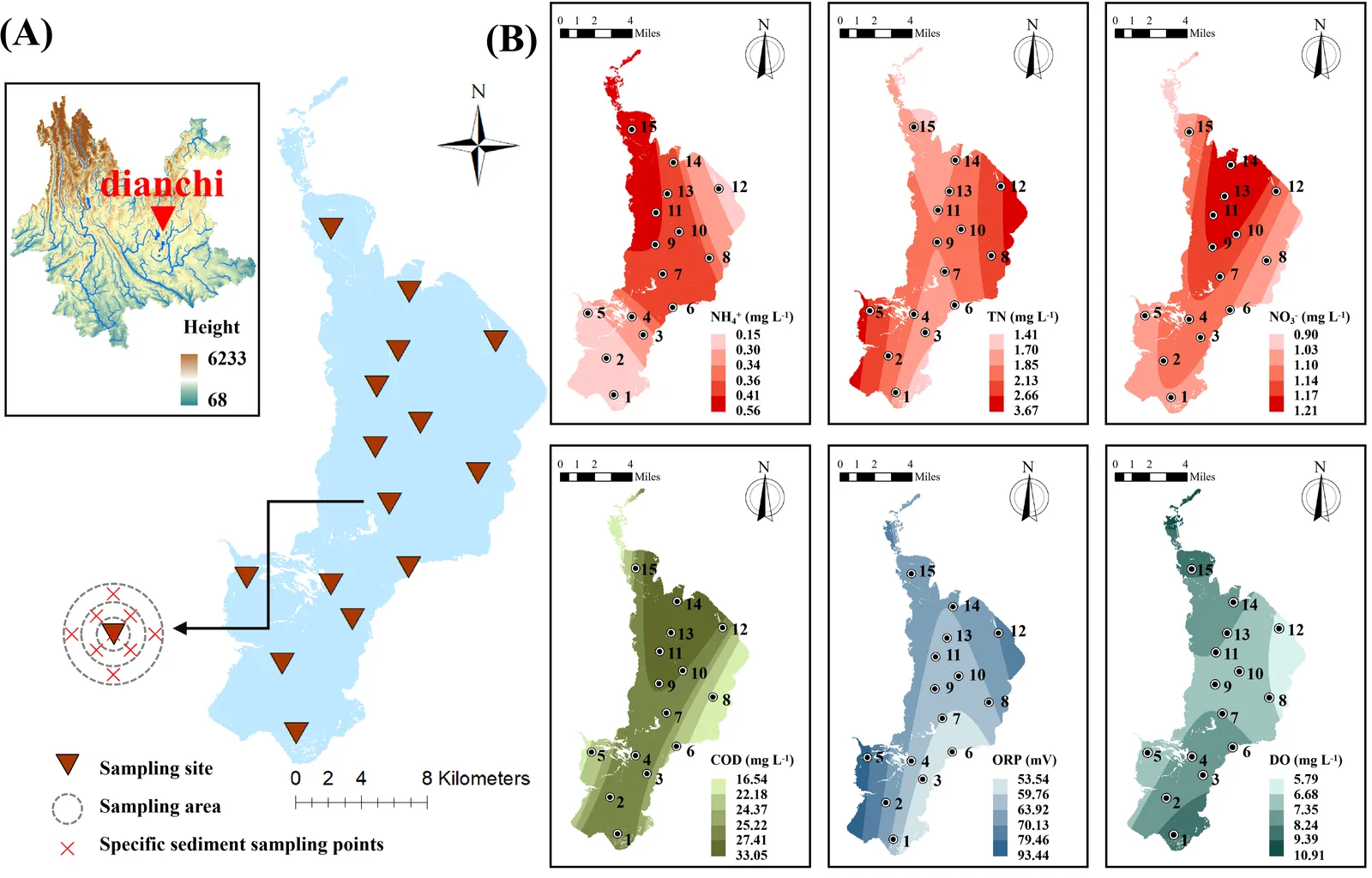
Geographical information and water quality of Dianchi Lake. (**A**) Geographical location and sampling site distribution of Dianchi Lake. (**B**) Distributions of sampling points and concentration gradients of NH₄^+^, TN, NO**₃**^−^, COD, ORP, and DO.

Sediment analysis was conducted in Dianchi Lake (102°42'31"E, 25°02'11"N), Yunnan Province, China, with a focus on iron-respiring bacteria, as well as carbon and nitrogen cycles. Sampling was performed in August 2024 (Fig. S1), when Dianchi Lake was still experiencing prominent pollution issues, including eutrophication. We uniformly selected 15 sampling sites as centers (Table S1). For each sampling center, 1, 4, and 4 specific sampling points are selected within the radii of 1, 2, and 3 km, respectively, as shown in Fig. 1A. Sediment samples were taken from the 5–10 cm depth interval below the sediment–water interface of the sediment core, with a weight of approximately 0.16 kg. They were placed on dry ice immediately upon collection, transported to the laboratory, and stored at −80°C until subsequent analysis. Each sediment sample was thawed in an anaerobic glove box (filled with 96% nitrogen and 4% hydrogen; Coy Laboratory Products, USA), during which impurities such as gravel were manually removed. The sample was then homogenized by vigorous stirring with an electric stirrer for 15 min (Fig. S7).

Water samples were collected at the sediment–water interface using a water sampler. To avoid potential variations in certain parameters caused by water sample transport and storage, measurements of temperature, pH, electrical conductivity (EC), oxidation-reduction potential (ORP), turbidity (NTU), and dissolved oxygen (DO) were conducted on-site for water samples collected at each sampling point. Following these measurements, water samples were stored at 4°C and transported to the laboratory for analysis of additional physicochemical properties.

### Physicochemical analysis

At the sampling sites, water temperature, pH, and ORP were measured using a portable pH meter (PHB-4, Shanghai Leici, China). DO was measured with a DO meter (JPBJ-608, Shanghai Leici, China), EC with a conductivity meter (OHR-EC10, Hongrun, China), and turbidity with a turbidimeter (LH-Z10A, Luheng, China). The water sample was shaken to ensure homogeneity and then poured into a glass graduated cylinder. After standing for 1 h at room temperature in the dark, the supernatant was carefully collected using a pipette. The supernatant was filtered through a 0.45 µm membrane, and the concentrations of NH₄^+^, NO₃^−^, and total nitrogen (TN) in the water sample were determined using a spectrophotometer (HD-UV90, Holder). Chemical oxygen demand (COD) was determined via potassium dichromate titration using ammonium ferrous sulfate as the titrant. Total iron was determined by 1,10-phenanthroline spectrophotometry. For total dissolved solids (TDS), samples were placed in evaporation dishes, evaporated to dryness, treated with H₂O₂ to remove organic matter, dried, and weighed to calculate TDS.

### Electrochemical characterizations

The electron donating capacity (EDC) and electron accepting capacity (EAC) of the sediments were determined using mediated electrochemical analysis. A three-electrode system was employed, consisting of a glassy carbon working electrode, an Ag/AgCl reference electrode, and a platinum wire auxiliary electrode. All operations were performed in an anaerobic glove box. The collected sediment samples were grouped, thoroughly mixed, and suspended in an electrolyte solution of pH 7.1 containing 0.1 mol L⁻¹ KCl and 0.01 mol L⁻¹ MOPS buffer (12). The electrolyte was purged with N₂ for 30 min before use. The sediment loading was 0.1–3 mg, and the suspension was maintained by magnetic stirring at 400 rpm. EDC measurements were performed with 125 µmol L⁻¹ ABTS as the electron mediator, and EAC measurements with 125 µmol L⁻¹ ethyl viologen dibromide (EtV) as the electron mediator (13). The sediments were tested in chronoamperometric mode at applied potentials of 0.41 V and −0.69 V (vs Ag/AgCl). The total transferred electrons were quantified by integrating the current–time curves (14). Additionally, the blank electrolyte current was measured during the assay. The amount of transferred electrons, *n* (mol), was calculated using the following equation:


$$n=\frac{\int (I_{total}-I_{blank})dt}{F}$$

where *I*_total_ is the measured total current (µA), *I*_blank_ is the blank electrolyte current (µA), *t* is the measurement time (s), and *F* is the Faraday constant (*F* = 96,485 C mol^−1^).

### DNA extraction and metagenomic sequencing

This study employed paired-end 150 bp shotgun metagenomic sequencing. DNA was extracted from sediment samples using the Power Soil DNA Isolation Kit (Mo Bio Laboratories, Carlsbad, CA, USA). Following genomic DNA extraction, the DNA was examined by 1% agarose gel electrophoresis and subsequently fragmented to approximately 350 bp using a Covaris M220 instrument. Libraries were prepared using the NEXTFLEX Rapid DNA-Seq Kit (Bioo Scientific, USA), with ligation of the “Y-shaped” adapters. Adapter self-ligation products were removed by magnetic bead selection, and the libraries were enriched by PCR amplification. The final library was obtained by magnetic bead recovery of the PCR products. The library DNA was circularized into single-stranded circles, non-circularized sequences were removed, and the product was purified to generate DNA nanoballs. Sequencing was performed on the DNBSEQ-T7 platform using the NBSEQ-T7RS Reagent Kit (FCL PE150) version 3.0 (Table S9).

### Gene assembly construction and function set annotation

Gene prediction was performed on the assembled contigs using Prodigal v2.6.3. CD-HIT v4.6.1 (http://www.bioinformatics.org/cd-hit/) was employed with a 90% sequence identity threshold for clustering, and the longest sequence from each cluster was taken as the representative to construct the non-redundant gene set. The non-redundant gene set was aligned against the NR database (version of October 7, 2024) and the KEGG database (version of October 7, 2024) (http://www.genome.jp/kegg/) using DIAMOND v2.0.13 (http://ab.inf.uni-tuebingen.de/software/diamond/) in BLASTP mode with an E-value ≤ 1e-5, yielding taxonomic and functional annotations. Gene abundance was normalized using the TPM method, which simultaneously corrects for gene length and sequencing depth to ensure comparability across samples. Ultimately, abundance profiles were constructed at each taxonomic level.

### Data processing and analysis

Kriging interpolation in ArcMap 10.8 was used to examine the distribution of environmental factors in Dianchi Lake. All subsequent statistical analyses were performed in R 4.5.0, and figures were generated using the ggplot2 package. Principal Coordinates Analysis (PCoA) based on Bray–Curtis distance was employed to visualize differences in microbial community structure, and permutational multivariate analysis of variance (PERMANOVA, 999 permutations) was performed using the adonis2 function in the vegan package to test the significance of differences between groups. Redundancy analysis (RDA) was used to explore the coupling relationships among carbon and nitrogen cycling genes, iron‑respiring bacteria abundance, and environmental factors. After diagnosis of variance inflation factor (VIF), forward selection was used to select significant environmental variables, and Monte Carlo permutation tests (999 permutations) were applied to assess the significance of the global model and constrained axes (*P* < 0.05). Pearson correlation analysis was used to construct molecular ecological networks. Based on genus‑level relative abundance, pairwise correlation coefficients were calculated; only associations with |*r*| > 0.6 and *P* < 0.05 were retained, and *P*‑values were corrected using the Benjamini–Hochberg method (FDR), with edges retained if corrected *P* < 0.05. Network visualization was performed in Gephi. Variation partitioning analysis (VPA) was conducted using the varpart function in the vegan package to partition the explained variation of gene structure by specified environmental factors, and the significance of each fraction was tested by permutation tests (anova.cca, 999 permutations) (*P* < 0.05). Structural equation modeling (SEM) was performed using the piecewiseSEM package to evaluate the direct and indirect effects of environmental factors on the abundance of carbon and nitrogen cycling genes. Model overall fit was assessed using Fisher's C test (*P* > 0.05 considered acceptable), and path coefficients were considered significant at *P* < 0.05. Heatmaps were generated by performing z‑score normalization on gene relative abundances and visualized using the ComplexHeatmap package.

## RESULTS

### Environmental factors

Dianchi Lake exhibited eutrophication, with ammonia nitrogen concentrations being generally elevated. Ammonia nitrogen concentrations ranged from 0.24 ± 0.04 to 0.50 ± 0.08 mg/L, showing a gradual decreasing trend from north to south. Nitrate nitrogen displayed a similar spatial distribution pattern, with concentrations ranging from 1.47 ± 0.20 to 4.58 ± 0.95 mg/L. However, TN concentrations were higher in the eastern and southwestern regions (Fig. 1B). This was likely attributable to extensive agricultural activities in these areas, where non-point source pollution led to substantial inputs of organic nitrogen into the lake (15). Additionally, the long hydraulic retention time in these regions facilitated the deposition of particulate organic nitrogen. Regarding other environmental factors, the parameters of COD (20.7 ± 6.1–33.2 ± 4.3 mg/L), TDS (216 ± 28–437 ± 33 mg/L), and EC (1347 ± 37–2740 ± 68 μS/cm) exhibited relatively consistent spatial distributions, all showing a gradual decreasing trend from north to south. Iron ion concentrations were generally below 0.6 mg/L, with relatively higher values observed in the southern area. Notably, DO and ORP displayed unique spatial distribution characteristics. ORP was relatively high in the southwestern part of Dianchi Lake, reaching 91.50 ± 5.71 mV, while higher DO concentrations, up to 10.2 ± 1.5 mg/L, were mainly concentrated in the northern and southern parts of the lake. Furthermore, the pH of Dianchi Lake was relatively uniformly distributed, remaining consistently around 8.5 (Fig. S2; Table S2).

### Microbial community structure

**Fig 2:**
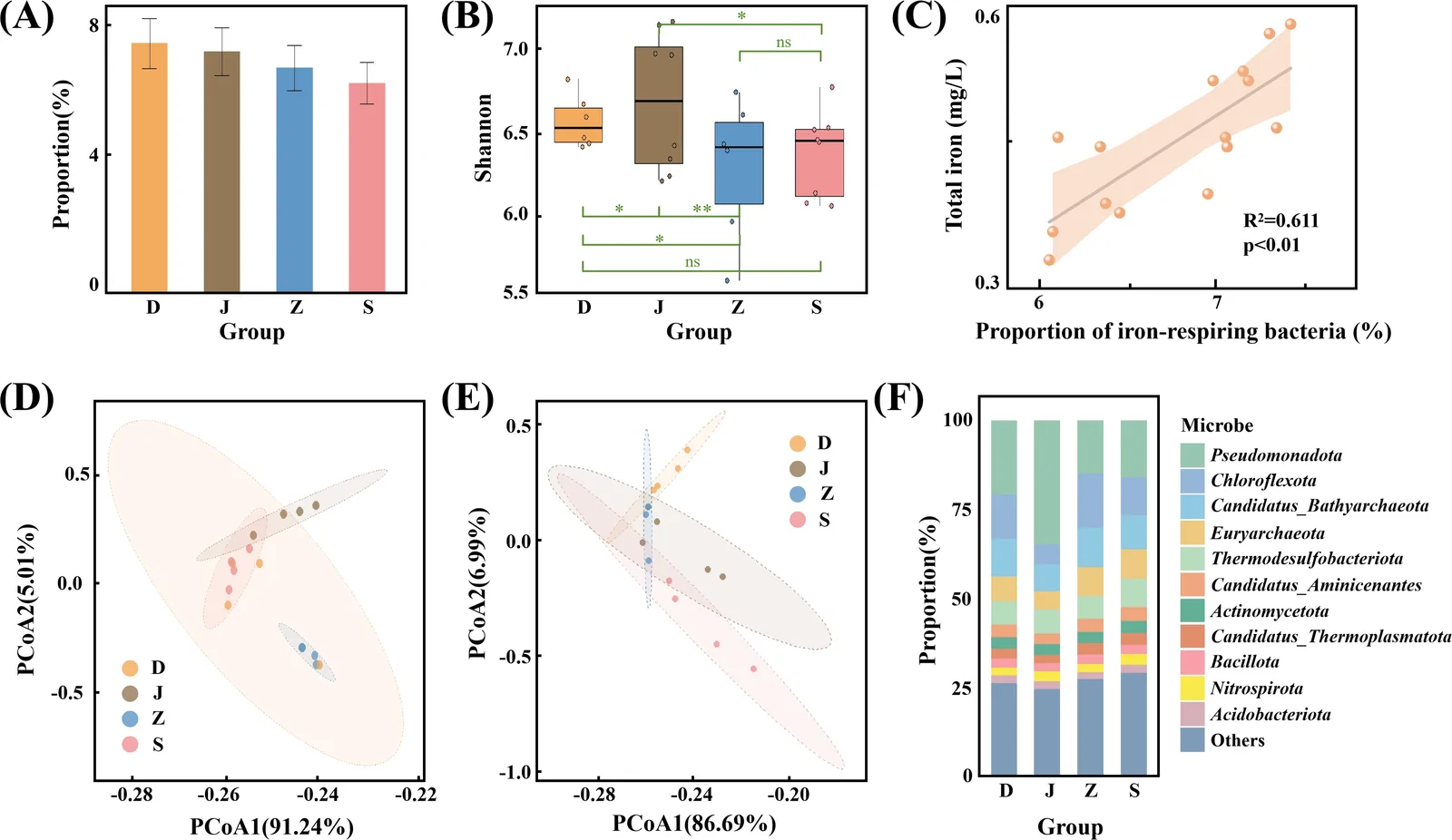
The microbial community structure of the sediment. (**A**) The proportions of iron-respiring bacteria in groups. (**B**) Shannon index of each group. ***, *P* < 0.001; **, *P* < 0.01; *, *P* < 0.05; ns, not significant. (**C**) Fitting of iron-respiring bacteria abundance and iron concentration. (**D**) PCoA of the overall microbial community structure. (**E**) PCoA of iron-respiring bacterial community structure. (**F**) Microbial population abundance on the phylum level.

Metagenomic sequencing analysis revealed that the predominant microbial populations at the phylum level were *Pseudomonadota* (21.77%), *Chloroflexota* (10.83%), *Candidatus* Bathyarchaeota (9.51%), *Euryarchaeota* (7.07%), and *Thermodesulfobacteriota* (6.97%) (Fig. 2F; Fig. S3). Among these, *Candidatus* Bathyarchaeota and *Euryarchaeota* were the main archaeal populations in the sediments and have been confirmed to be closely associated with the carbon cycle (16). Notably, the relative abundance of *Pseudomonadota* was significantly influenced by environmental factors, exhibiting a variation range of up to 33.21%, and this phylum represents a key microbial group in nitrogen fixation and nitrification pathways (17).

The species of iron-respiring bacteria identified are listed in Table S6. The sediments harbored a high abundance of iron-respiring bacteria (Fig. 2A), with a total abundance accounting for 6.79% of the overall microbial community. Correlation analysis indicated a strong positive correlation between their abundance and iron concentrations (Fig. 2C), consistent with previous research findings (18). Based on the abundance of iron-respiring bacteria, the sampling sites were categorized into four groups (D, J, Z, and S) in descending order (Table S3). The four groups showed significant differences in community structure (Fig. 2D and E), which could be evident from the α-diversity. Group J exhibited a relatively high Shannon index, suggesting greater species richness and diversity (Fig. 2B). In contrast, group Z showed the lowest Shannon index, potentially indicating environmental stress on the community (Table S4). The relative abundance of *Pseudomonadota* in group Z decreased to 13.15%, which may have constrained nitrogen fixation and nitrification pathways within the nitrogen cycle.

### Carbon cycling genes

**Fig 3:**
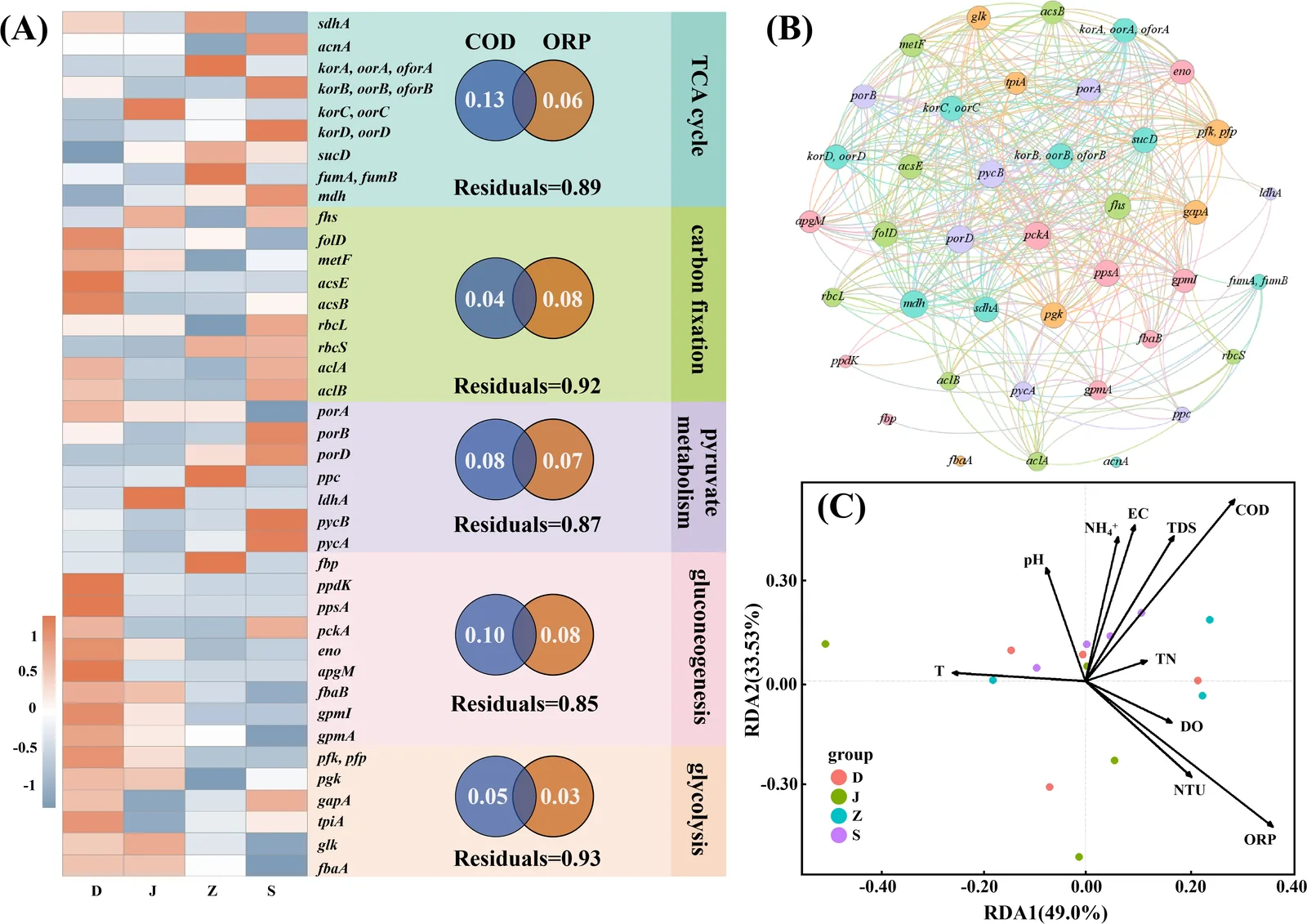
Characteristics of carbon cycling genes in different environments. (**A**) Gene abundance of carbon cycle and VPA displaying effects of COD and ORP on carbon cycle. (**B**) Co-occurrence network of carbon cycling genes, and (**C**) RDA showing the shifts in the pattern of carbon cycling genes as affected by environmental factors.

In the analysis of carbon cycling genes, this study primarily focused on the tricarboxylic acid (TCA) cycle, carbon fixation, pyruvate metabolism, gluconeogenesis, and glycolysis. Heatmap analysis of gene abundances revealed high abundances of carbon fixation, gluconeogenesis, and glycolysis genes in group D (Fig. 3A; Table S12). This may reflect an adaptive response of microorganisms to the low COD concentrations in these areas, necessitating more efficient carbon acquisition and metabolism for survival. Conversely, the module with the highest relative gene abundance was pyruvate metabolism in group S, which is associated with amino acid synthesis and energy production, indicating a focus on the utilization of carbon sources (19). Regarding the interconnections among genes within the carbon cycle, co-occurrence network analysis revealed considerable correlations among the various modules (Fig. 3B; Table S11). Key nodes influencing the interactions between modules included the iron-sulfur protein methyltransferase *apgM*, methanol dehydrogenase *mhd*, and the pyruvate ferredoxin oxidoreductase alpha subunit *porA*. Furthermore, VPA was employed to assess the impact of environmental factors on carbon cycling genes. COD and ORP were identified as the two most influential factors (Fig. 3A; Fig. S8). Specifically, COD exerted a significantly stronger effect on the TCA cycle (0.13) compared to ORP (0.06), whereas the opposite trend was observed for the carbon fixation and pyruvate metabolism modules. Notably, the carbon cycling genes in group S exhibited a greater response to nitrogen and COD concentrations than those in the other samples, while the responses of genes in the remaining samples to environmental factors were relatively consistent (Fig. 3C).

### Nitrogen cycling genes

**Fig 4:**
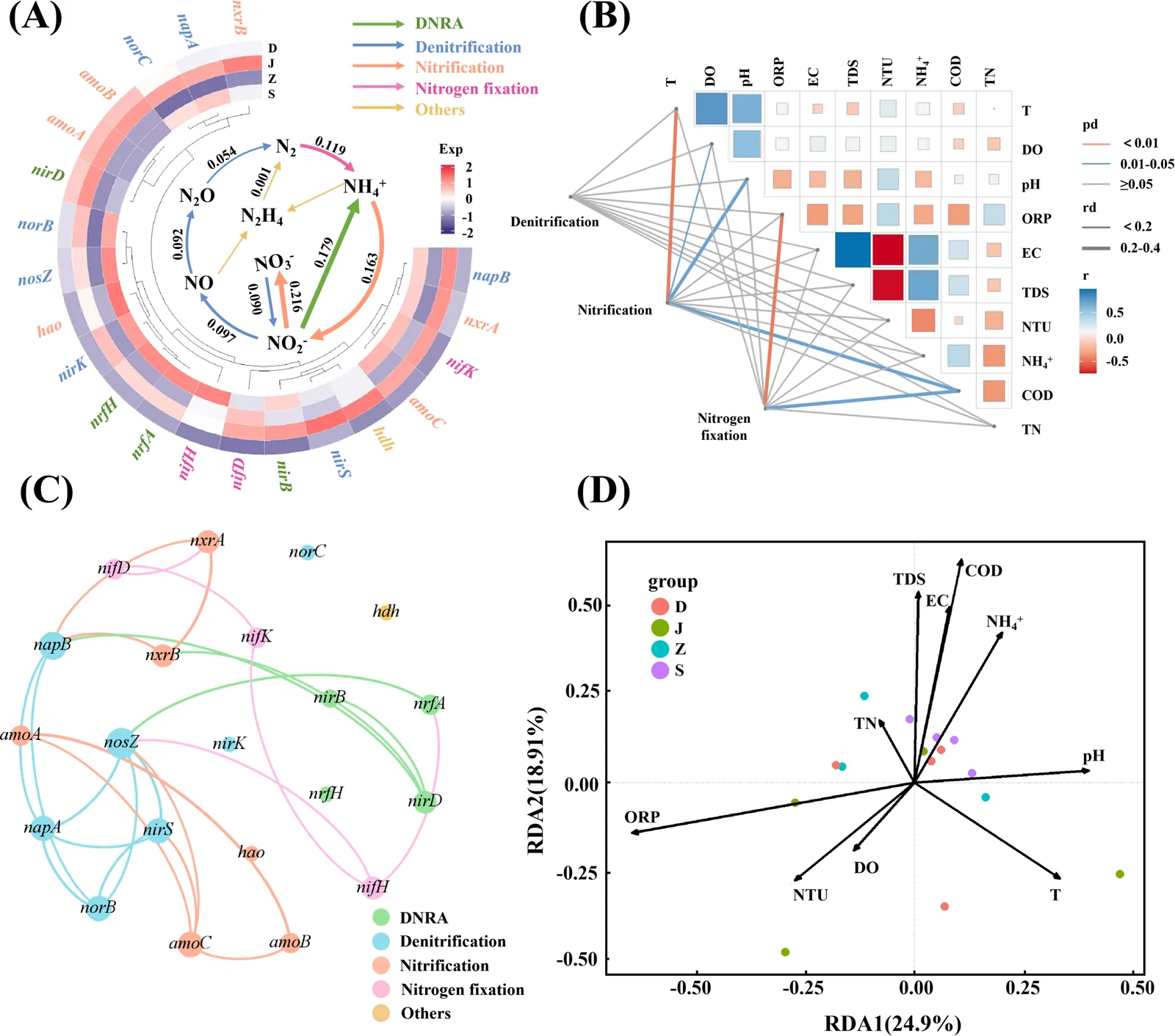
Characteristics of nitrogen cycling genes in different environments. (**A**) Gene abundance of nitrogen metabolism and a model map of the nitrogen cycle. The thickness of the arrow represents the proportion of gene abundance. (**B**) Pearson’s correlation matrix of environmental variables and their relationships with nitrogen cycling functional genes, analyzed using Mantel tests. (**C**) Co-occurrence network of nitrogen cycling genes, and (**D**) RDA showing the shifts in the patterns of nitrogen cycling genes as affected by environmental factors.

We annotated 21 key genes involved in the nitrogen cycle, including seven genes belonging to the denitrification module, six to the nitrification module, four to the dissimilatory nitrate reduction to ammonium (DNRA) module, and genes from other nitrogen cycling pathways (Table S5). The heatmap showed higher abundances of nitrogen cycling genes in group J (Fig. 4A), where the corresponding microbial populations exhibited higher Shannon indices, suggesting that microorganisms related to the nitrogen cycle gained more living space under the environmental conditions of group J. Notably, nitrification genes such as *amoA* and *amoB* were significantly more abundant in group D, whereas group S was enriched with denitrification genes (e.g., *nirK*) and DNRA genes (e.g., *nrfH*), suggesting potential dynamic regulation among pathways within the nitrogen cycle. From a holistic perspective of the Dianchi Lake sediment nitrogen cycle, nitrification and DNRA exhibited the highest metabolic potential, which may explain the substantial proportions of NH₄^+^ and NO₃^−^ in TN. Co-occurrence network analysis revealed strong connectivity between the denitrification module and other modules, particularly with the DNRA and nitrification modules (Fig. 4C). Strong connections were observed between the denitrification module and other modules, as well as between DNRA and nitrification. The nitrate reductase gene *napB* emerged as a key node, forming connections with *amoC* (nitrification), *nrfA* (DNRA), and *nifH* (nitrogen fixation). Additionally, the network indicated that the nitrogen fixation module was relatively independent, showing weak associations with the other modules. Pearson correlation analysis assessing the relationships between nitrogen cycling and environmental factors revealed a significant association between nitrogen metabolism and COD (Fig. 4B; Table S10), which was consistent with RDA results. Interestingly, we found that the abundance of nitrogen cycling genes in group S was most strongly influenced by COD and NH₄^+^, whereas the effects were smaller in groups D and J (Fig. 4D), suggesting that the degree of carbon-nitrogen coupling was closely related to environmental factors.

## DISCUSSION

### Effects of iron-respiring bacteria on communities

We observed that the abundance of iron-respiring bacteria varied across different regions, with a maximum difference of 1.37% in their relative abundance. Notably, in areas where iron-respiring bacteria were highly active, such as groups D and J, the Shannon index was relatively higher, indicating greater species diversity. This suggests that iron-respiring bacteria may employ multiple mechanisms to regulate microbial community structure. First, iron-respiring bacteria may shape community structure through competition (20). In groups Z and S, the abundance of iron-respiring bacteria was relatively low owing to the influence of iron ion concentration. Concurrently, we observed a marked increase in the abundance of *Candidatus* Thermoplasmatota, which displayed a trend opposite to that of the iron-respiring bacteria. This archaeal group is known to live primarily by metabolizing organic matter, and recent metagenomic studies have revealed that some novel lineages within the phylum Thermoplasmatota possess metabolic pathways for degrading complex organic carbon and encode key genes for the utilization of small-molecule organic substrates such as acetate (21). This substantially overlaps with the large amounts of carbon sources, such as acetate and other electron donors, required by iron-respiring bacteria for their metabolism. In the field of anaerobic microbial ecology, several classic studies have demonstrated, through kinetic experiments and field observations, that intense substrate competition exists between iron-respiring bacteria and other heterotrophic groups, such as methanogens and sulfate-reducing bacteria. They compete for key electron donors such as H₂ and acetate, and iron-respiring bacteria often prevail because of their higher substrate affinity, thereby restricting the expansion of competing populations (22). Additionally, iron-respiring bacteria engage in various symbiotic relationships that can enhance the abundance of certain microorganisms in the sediments, particularly those that lack competitiveness under the influence of environmental factors. Previous studies have reported symbiotic associations between iron-respiring bacteria and methanogens or *Firmicutes*, enabling them to collectively metabolize organic compounds that are recalcitrant to degradation by a single species (23). This may be attributed to the ability of iron-respiring bacteria to enhance the electron transfer capacity of the microbial community. This view is supported by studies showing that iron-respiring bacteria help the community metabolize recalcitrant intermediates, such as long-chain fatty acids, thereby driving the overall degradation process (24). Because Fe³^+^ offers thermodynamic advantages as an electron acceptor, iron-respiring bacteria exhibit high electron transfer efficiency, particularly in the presence of conductive materials such as magnetite (25). By constructing electron transfer networks based on these conductive materials, iron-respiring bacteria can achieve syntrophic metabolism with other microorganisms over long distances. In this study, we found that in group J, where iron-respiring bacteria were abundant, the abundance of *Acidobacteriota*, a group known for its ability to metabolize complex organic compounds, peaked, coinciding with high overall diversity. These observations are consistent with previous findings. Furthermore, research has found that the metabolism of iron-respiring bacteria can indirectly promote the survival of other populations because iron-respiring bacteria generate redox-active electron shuttles when transferring electrons to acceptors, which can then be utilized by other microorganisms (26). Moreover, a past study indicated that the chelating agents produced by iron-respiring bacteria can accelerate the degradation of organic matter, thereby facilitating the metabolic activities of other microorganisms (27).

### Carbon cycle

**Fig 5:**
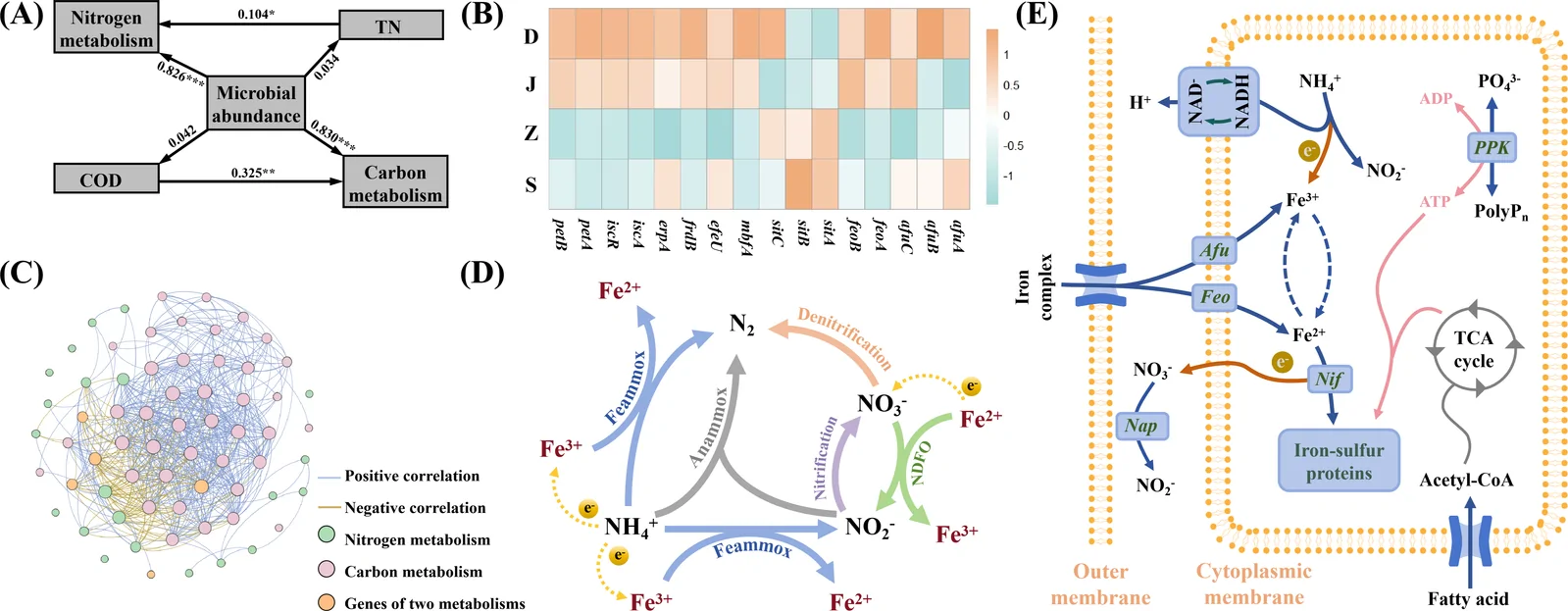
Analysis of carbon-nitrogen coupling. (**A**) Structural equation modeling displaying the relative influence of microbial abundance and environmental factors on carbon and nitrogen metabolism genes. ***, *P* < 0.001; **, *P* < 0.01; *, *P* < 0.05. (**B**) Heatmap of iron metabolism gene abundances across groups. (**C**) Co-occurrence network of carbon-nitrogen cycling genes. (**D**) Schematic diagram of the coupling between iron metabolism and nitrogen cycle, where NDFO stands for nitrate-dependent Fe(II) oxidation. (**E**) Genome-based metabolic model for iron-respiring bacteria.

**Fig 6:**
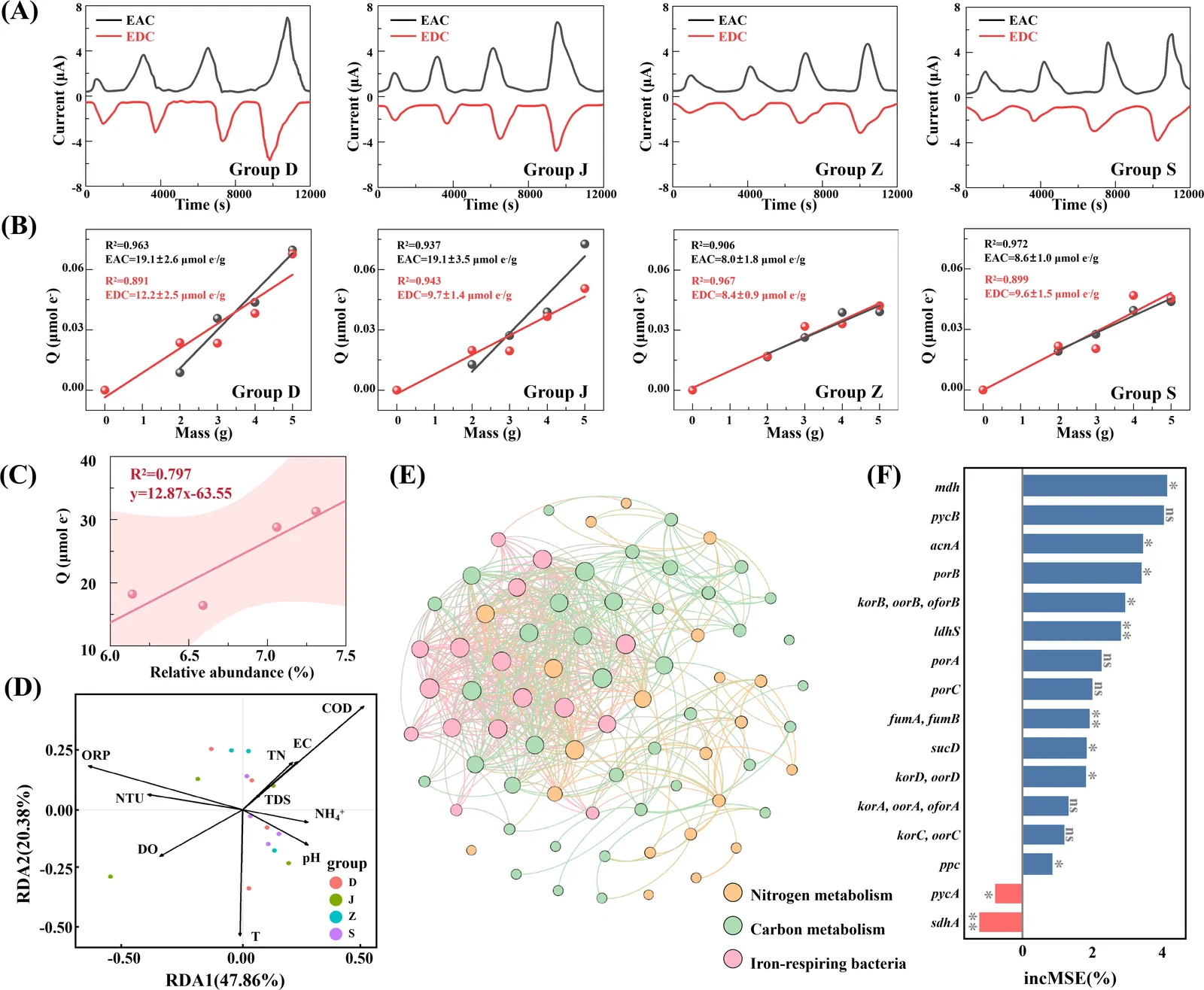
Relationship between iron-respiring bacteria and carbon-nitrogen cycling. (**A**) The reduction and oxidation current responses of each sediment group via mediated electrochemical reduction and mediated electrochemical oxidation. (**B**) Linear fitting of electron count versus mass for each group. (**C**) Linear fitting of EEC and iron-respiring bacteria abundance. (**D**) RDA showing the shifts in the patterns of iron-respiring bacteria as affected by environmental factors. (**E**) Co-occurrence network of iron-respiring bacteria and carbon-nitrogen cycling genes. (**F**) Multiple linear regression model of the TCA cycle and carbon fixation to iron-respiring bacteria. ***, *P* < 0.001; **, *P* < 0.01; *, *P* < 0.05; ns, not significant.

In terms of the carbon cycle, VPA revealed that the carbon cycle was sensitive to ORP and COD values. Studies have shown that ORP can alter the distribution of microbial communities, thereby directly influencing carbon transformation pathways (28). Under high-ORP conditions, microbial communities tend to generate CO₂ through pyruvate metabolism and the TCA cycle. Conversely, under low-ORP conditions, communities undergo fermentation or anaerobic respiration, emitting CH₄ (29). ORP reflects the electron transfer environment, indicating that changes in carbon cycling pathways largely depend on the availability of electron donors and acceptors in the water body. Iron-oxidizing bacteria can generate the electron acceptor Fe³^+^, thereby increasing ORP, whereas iron-reducing bacteria have the opposite effect (30). In this study, the abundance of iron-oxidizing bacteria was slightly higher than that of iron-reducing bacteria. Therefore, collectively, iron-respiring bacteria in Dianchi Lake increased environmental ORP, thereby influencing carbon cycling pathways. RDA revealed a close relationship between iron-respiring bacteria and COD (Fig. 6D), which was predominantly positive. Organic matter serves as a crucial electron donor for the metabolism of iron-respiring bacteria, supporting their survival. A study demonstrated that the iron-respiring bacterium *Candidatus Methanoperedens nitroreducens*, which was detected in this study, contains numerous genes encoding multi-heme c-type cytochromes (31). This bacterium can utilize methane as an electron donor, directly oxidizing it to CO₂ to obtain energy for survival. Notably, in groups Z and S, where the abundance of iron-respiring bacteria was limited, some genes belonging to the TCA cycle and pyruvate metabolism were relatively abundant. Multiple linear regression analysis indicated a strong link between these modules and iron-respiring bacteria, with *mdh* being the carbon cycle gene exerting the greatest influence on iron-respiring bacteria (Fig. 6F). This may be attributed to the potent electron transfer capacity and numerous symbiotic relationships of iron-respiring bacteria (32), allowing their influence on microbial community distribution to extend to carbon cycling processes. The structural equation model analysis demonstrated that iron-respiring bacteria can establish connections with carbon cycling genes through microbial communities (Fig. 5A). The metabolic activity of iron-respiring bacteria on recalcitrant organic compounds benefits carbon-metabolizing microorganisms, and the subsequent growth of microbial communities further enhances organic matter metabolism, enriching carbon cycling genes. In this study, group D, which harbored abundant iron-respiring bacteria, exhibited remarkably strong carbon cycling genes despite being located in an area with limited COD availability. The microbial community in group D demonstrated the highest metabolic potential for COD, potentially benefiting from the symbiotic effects of iron-respiring bacteria. Therefore, iron-respiring bacteria may have multiple promoting effects on the carbon cycle.

### Nitrogen cycle

In terms of the nitrogen cycle, our study found that DNRA and nitrification genes were relatively abundant, and they may be the main pathways of the nitrogen cycle. In areas with low iron-respiring bacteria abundance, such as group S, the abundance of *nrf* family genes reached its peak here, indicating a greater potential for microbial DNRA. A recent study indicated that when their metabolism is inhibited, iron-respiring bacteria may shift to alternative metabolisms, accompanied by an increase in genes associated with the carbon, nitrogen, or sulfur cycles (33). This suggests that switching electron donors or acceptors is a viable survival strategy for iron-respiring bacteria, enabling them to complete metabolism using available substances in their surroundings. Previous studies have demonstrated that iron-reducing bacteria possess the capacity for ammonia oxidation, a process termed Feammox (34). In areas with high ammonium nitrogen concentrations, iron-reducing bacteria can utilize NH₄^+^ as an electron donor to reduce Fe³^+^. Heatmap analysis revealed that the *sit* gene encoding the iron transport system was highly abundant in iron-respiring bacteria in group S (Fig. 5B; Fig. S9). Meanwhile, enrichment of nitrification-related functional genes such as *amoC*, *nxrA,* and *hao* was also detected in group S. Previous studies have confirmed that *amo* and *hao* are significantly enriched in Feammox enrichment cultures (35), indicating the presence of an active ammonium oxidation process in this system. The group S environment is characterized by high ammonium concentrations and an abundance of DNRA genes, which can promote ammonium production, thereby providing the substrate conditions for a coupled ammonium oxidation–iron reduction process. The high abundance of the *sit* gene may indirectly support iron reduction at the substrate supply level by enhancing the iron acquisition capacity of iron-respiring bacteria. However, this inference remains to be experimentally verified. A recent study demonstrated a synergistic mechanism between Feammox and DNRA (36), which aligns with the findings of our study. These two reactions, which respectively generated and consumed NH₄^+^, formed a dynamic equilibrium. We hypothesize that if iron-respiring bacteria were to encounter a more suitable living environment, this balance could be disrupted, potentially alleviating eutrophication. In areas with a relatively higher abundance of iron-respiring bacteria, the *nrf* family genes gradually lost their dominance and were supplanted by denitrification and nitrification, represented by the *nap* and *amo* family genes. Furthermore, in these areas, the abundance of nitrogen cycle genes was relatively balanced. This may be attributed to increased community diversification, wherein various nitrogen-cycling microorganisms have secured suitable ecological niches. Notably, iron-oxidizing bacteria capable of participating in nitrate-dependent ferrous iron oxidation, such as *Rhodanobacter* and *Thermomonas*, were observed. Studies have demonstrated that these bacteria utilize NO₃^−^ as an electron acceptor, producing NO₂^−^ or N₂ (37), thereby enhancing denitrification reactions. Iron-reducing bacteria and iron-oxidizing bacteria exert distinctly different effects on nitrogen pathways, collectively coupling the nitrogen cycle and promoting its dynamic cycling (Fig. 5D). The co-occurrence network diagram revealed close interconnections among internal nitrogen cycling pathways, demonstrating the coupling relationships among various modules. Iron-respiring bacteria not only directly metabolize nitrogen elements but also influence microbial community structure. Therefore, iron-respiring bacteria represent a crucial factor in controlling nitrogen pollution in sediments.

### Coupling of carbon and nitrogen cycles

In sediment biogeochemical cycles, the reactions of different elements do not occur independently, and the pathways at each point are quite different (Fig. S4 and S5). Through the co-occurrence network analysis of carbon and nitrogen cycling genes, we found that they exhibited predominantly positive correlations, with a positive-to-negative correlation ratio of approximately 3 (Fig. 5C). Analysis of the nodal genes revealed that coupling arises, in part, from the cross-utilization of intermediate products (Table S7). For instance, *glnA* and *gdhA* exhibited close linkages with other cycling pathways. They have been demonstrated to consume α-ketoglutarate, a crucial intermediate in the TCA cycle, during the catalysis of glutamate synthesis from ammonia and glutamine (38). Upon incorporating iron-respiring bacteria into the co-occurrence network, we found that they were tightly connected to carbon and nitrogen metabolic genes and occupied central nodal positions within the network (Fig. 6E). This may be because the functions of iron-respiring bacteria intersect with those of certain cycling intermediates. The electron shuttles produced by iron-respiring bacteria can serve as mobile electron donors, supplementing the electrons required for the metabolism of other microorganisms (39). Such shuttles act as universal metabolic intermediates, effectively compensating for the missing links in electron transfer chains caused by spatial segregation of microorganisms within the reaction system. Through mediated electrochemical analysis, the EDC was found to range from 8.4 to 12.2 μmol e^–^/g, with groups D and J exhibiting significantly higher values than the other groups. The EAC followed the same pattern, with the lowest value of 8 μmol e^–^/g being observed in group Z (Fig. 6A and B; Table S8). This trend was consistent with the distribution of iron-respiring bacteria (Fig. 6C), suggesting that their presence enhanced the overall electron exchange capacity (EEC) of the sediment. A recent study has shown that short-range microbial electron transfer activities in sediments can interconnect, thereby establishing long-distance electron transfer pathways (12). Therefore, the abundance of iron-respiring bacteria facilitated the construction of longer-distance electron transfer pathways. Furthermore, the metabolism of iron-respiring bacteria can decompose recalcitrant intermediate products, thereby facilitating overall metabolic progress. In the absence of such intermediate processing, accumulation may occur, leading to pollution or even toxicity, while microbial communities downstream in the metabolic network may perish due to an inability to obtain energy. Additionally, iron-respiring bacteria themselves possess multiple metabolic pathways, enabling them to directly participate in the carbon and nitrogen cycles (Fig. 5E). Studies have demonstrated that iron-respiring bacteria can utilize NO₃^−^ and NO₂^−^ as electron acceptors, as well as organic matter and NH₄^+^ as electron donors for metabolism. Notably, through co-occurrence network analysis, we found that *ureC* was one of the key coupling nodes. It catalyzes the decomposition of urea into ammonia and CO₂, leading to changes in carbon and nitrogen contents in the water body (40). Dianchi Lake was rich in organic nitrogen, which could be utilized by iron-respiring bacteria as electron donors. If iron-respiring bacteria metabolically decomposed organic nitrogen, this would simultaneously drive the release of carbon and nitrogen elements, thereby promoting both cycles. From the perspective of heavy metal pollution, iron-respiring bacteria are also of great significance to the overall aquatic biogeochemical cycles (41). During the dissolution of iron minerals, iron-respiring bacteria can release adsorbed heavy metal ions, leading to toxicity dispersion. Meanwhile, iron-respiring bacteria can also utilize heavy metal ions as terminal electron acceptors. Upon reduction, these ions often exhibit decreased solubility and form precipitates, thereby immobilizing pollutants (42). Therefore, the activities of iron-respiring bacteria couple the carbon and nitrogen cycles (Fig. S6), representing a critical aspect that cannot be overlooked in the remediation of lake sediments.

### Conclusion

This study reveals that iron-respiring bacteria play an important role in carbon and nitrogen cycles in lake sediments. The results indicate that iron-respiring bacteria may be directly involved in carbon and nitrogen metabolism and that their selection of electron donors or acceptors is influenced by environmental factors. In addition, potential symbiotic relationships exist between iron-respiring bacteria and various other microorganisms. Iron-respiring bacteria may enhance the electron transfer and metabolic capacity of the microbial community, thereby altering the community structure and material cycling in the sediment. The metabolism of iron-respiring bacteria may directly or indirectly enrich certain carbon and nitrogen cycling genes, potentially serving to couple carbon and nitrogen cycles in the sediment. This study investigated the dynamic processes of carbon and nitrogen cycling in the lake and attempted to elucidate, from an electron-centric perspective, the metabolic mechanisms of iron-respiring bacteria related to carbon and nitrogen cycling. These findings are of considerable significance for future exploration of sediment evolution and lake remediation.

## SUPPLEMENTAL MATERIAL

Supplemental material aem.01007-26-s0001.docx Tables S1 to S12; Fig. S1 to S9.

ASM does not own the copyrights to Supplemental Material that may be linked to, or accessed through, an article. The authors have granted ASM a non-exclusive, world-wide license to publish the Supplemental Material files. Please contact the corresponding author directly for reuse.
