# Supplementary material for: Iron-respiring bacteria mediate regulatory mechanisms and pathways of electron donors and acceptors in carbon and nitrogen cycles
Source: Appl Environ Microbiol. 2026 Jul 22;92(8):e01007-26. doi: 10.1128/aem.01007-26 (PMC13488355; doi:10.1128/aem.01007-26)
Supplement: Supplementary file 1 [file aem.01007-26-s0001.docx]

**Supplementary Materials**

Iron-respiring bacteria mediate regulatory mechanisms and pathways of electron donors and acceptors in carbon and nitrogen cycles

**Table S1.** Coordinates of sampling sites.

| Sampling sites | Longitude (°E) | Latitude (°N) |
| --- | --- | --- |
| 1 | 102.65 | 24.69 |
| 2 | 102.64 | 24.72 |
| 3 | 102.68 | 24.74 |
| 4 | 102.62 | 24.76 |
| 5 | 102.67 | 24.76 |
| 6 | 102.71 | 24.77 |
| 7 | 102.70 | 24.80 |
| 8 | 102.75 | 24.82 |
| 9 | 102.69 | 24.83 |
| 10 | 102.71 | 24.85 |
| 11 | 102.69 | 24.86 |
| 12 | 102.76 | 24.89 |
| 13 | 102.70 | 24.88 |
| 14 | 102.71 | 24.91 |
| 15 | 102.66 | 24.94 |

**Table S2.** Environmental factors of sampling sites.

| Sampling sites | NH_4_^+^ (mg/L) | TN (mg/L) | COD (mg/L) | NO**₃**^-^  (mg/L) | ORP (mV) | DO (mg/L) | T (℃) | pH | EC (μS/cm) | TDS (mg/L) | **Turbidity (**NTU) | **Total iron**  (mg/L) |
| --- | --- | --- | --- | --- | --- | --- | --- | --- | --- | --- | --- | --- |
| 1 | 0.24 | 1.98 | 30.41 | 1.07 | 51.3 | 8.94 | 22.1 | 8.81 | 546 | 267 | 53.7 | 0.58 |
| 2 | 0.24 | 1.69 | 28.22 | 1.15 | 77.2 | 7.33 | 18.6 | 8.49 | 368 | 213 | 85.8 | 0.52 |
| 3 | 0.32 | 1.79 | 16.25 | 1.12 | 84.6 | 8.65 | 20.9 | 8.03 | 631 | 294 | 74.9 | 0.53 |
| 4 | 0.26 | 2.85 | 20.19 | 1.04 | 78.8 | 7.25 | 20.2 | 8.44 | 609 | 293 | 27.2 | 0.57 |
| 5 | 0.33 | 1.88 | 30.19 | 1.19 | 58.9 | 6.65 | 17.1 | 8.06 | 800 | 350 | 33.4 | 0.52 |
| 6 | 0.33 | 1.77 | 22.43 | 1.06 | 52.2 | 7.34 | 20.6 | 8.43 | 751 | 295 | 34.9 | 0.46 |
| 7 | 0.43 | 2.19 | 28.42 | 1.26 | 61.8 | 8.13 | 19.8 | 8.32 | 1070 | 422 | 30.1 | 0.45 |
| 8 | 0.35 | 2.31 | 25.70 | 1.04 | 63.4 | 7.07 | 16.8 | 8.06 | 795 | 337 | 31.3 | 0.40 |
| 9 | 0.47 | 1.67 | 33.27 | 1.07 | 72.0 | 6.69 | 19.9 | 7.88 | 660 | 305 | 56.8 | 0.33 |
| 10 | 0.37 | 1.79 | 24.49 | 1.24 | 44.4 | 6.28 | 16.7 | 8.05 | 785 | 353 | 28.2 | 0.38 |
| 11 | 0.50 | 1.71 | 29.19 | 1.19 | 54.8 | 7.82 | 19.9 | 8.23 | 1040 | 434 | 18.4 | 0.46 |
| 12 | 0.33 | 2.59 | 24.60 | 1.18 | 76.7 | 6.27 | 15.7 | 7.35 | 890 | 377 | 17.8 | 0.45 |
| 13 | 0.42 | 2.12 | 33.16 | 1.12 | 61.2 | 8.18 | 19.4 | 8.28 | 1080 | 429 | 29.6 | 0.39 |
| 14 | 0.27 | 1.88 | 28.53 | 1.16 | 72.4 | 7.84 | 19.2 | 8.19 | 1065 | 418 | 15.8 | 0.47 |
| 15 | 0.50 | 1.69 | 25.70 | 1.12 | 67.2 | 8.07 | 20.2 | 8.24 | 1060 | 429 | 23.1 | 0.36 |

**Table S3.** Grouping of sampling sites.

| Group | Sampling sites | Average proportion of **iron-respiring bacteria** |
| --- | --- | --- |
| D | 1, 2, 4, 14 | 7.31% |
| J | 3, 5, 6, 12 | 7.06% |
| Z | 8, 10, 13 | 6.59% |
| S | 7, 9, 11, 15 | 6.14% |

**Table S4.** Microbial diversity indexes of sampling sites.

| Sampling sites | sobs | ACE | Chao | Shannon | Simpson |
| --- | --- | --- | --- | --- | --- |
| 1 | 34554.0 | 34558.19779 | 34554.06349 | 6.685352 | 0.013453 |
| 2 | 36260.5 | 36266.26525 | 36260.56667 | 6.528024 | 0.061957 |
| 3 | 29851.0 | 29854.33312 | 29851.05716 | 6.241907 | 0.019386 |
| 4 | 37877.0 | 37881.56178 | 37877.04110 | 6.659951 | 0.012761 |
| 5 | 36811.5 | 36815.21947 | 36811.53100 | 6.981917 | 0.004971 |
| 6 | 36277.5 | 36279.23053 | 36277.50656 | 7.169818 | 0.004337 |
| 7 | 38356.5 | 38359.96428 | 38356.52863 | 6.083404 | 0.030857 |
| 8 | 35857.5 | 35862.29392 | 35857.56501 | 5.804898 | 0.042979 |
| 9 | 35588.0 | 35590.69922 | 35588.01351 | 6.624315 | 0.012640 |
| 10 | 36896.0 | 36900.40608 | 36896.04503 | 6.602467 | 0.011429 |
| 11 | 36958.0 | 36963.52379 | 36958.06891 | 6.390898 | 0.009489 |
| 12 | 37121.0 | 37125.32978 | 37121.02919 | 6.396841 | 0.010726 |
| 13 | 37759.0 | 37762.02804 | 37759.02157 | 6.516077 | 0.015847 |
| 14 | 39084.0 | 39084.43379 | 39084.00130 | 6.431275 | 0.023306 |
| 15 | 38072.5 | 38073.35958 | 38072.50255 | 6.507833 | 0.008144 |

**Table S5.** Genes involved in each process of the nitrogen cycle.

| Process | KEGG name |
| --- | --- |
| DNRA | *nrfA, nrfH, nirB, nirD* |
| Denitrification | *nosZ* |
|  | *norB, norC* |
|  | *nirS, nirK* |
|  | *napA, napB* |
| Nitrification | *amoA, amoB, amoC, hao* |
|  | *nxrA, nxrB* |
| Nitrogen fixation | *nifD, nifH, nifK* |
| Others | *hdh* |

**Table S6.** Genus and species of iron-respiring bacteria. Iron-respiring bacteria with a relative abundance exceeding 0.1% were selected.

| Genus | Species | Function |
| --- | --- | --- |
| *g__Thiobacillus* | *s__Thiobacillus_sp.* | Oxidation |
| *g__Dechloromonas* | *s__Dechloromonas_sp.* | Oxidation |
| *g__Rhodoferax* | *s__Rhodoferax_sp.* | Reduction |
| *g__Dechloromonas* | *s__Dechloromonas_sp._H13* | Oxidation |
| *g__Dechloromonas* | *s__Candidatus_Dechloromonas_phosphorivorans* | Oxidation |
| *g__Desulfuromonas* | *s__Desulfuromonas_sp.* | Reduction |
| *g__Dechloromonas* | *s__Candidatus_Dechloromonas_phosphoritropha* | Oxidation |
| *g__Rhodobacter* | *s__Rhodobacter_sp.* | Oxidation |
| *g__Gallionella* | *s__Gallionella_sp.* | Oxidation |
| *g__Desulfobulbus* | *s__Desulfobulbus_sp.* | Reduction |
| *g__Anaeromyxobacter* | *s__Anaeromyxobacter_sp.* | Reduction |
| *g__Pseudomonas* | *s__Pseudomonas_sp.* | Reduction |
| *g__Geobacter* | *s__Geobacter_sp.* | Reduction |
| *g__Dechloromonas* | *s__Dechloromonas_denitrificans* | Oxidation |
| *g__Azospira* | *s__Azospira_sp.* | Oxidation |
| *g__Thiobacillus* | *s__Thiobacillus_denitrificans* | Oxidation |

**Table S7.** Classification of gene in co-occurrence network analysis.

| Process | KEGG Name |
| --- | --- |
| Nitrogen metabolism | *nosZ, norB, norC, nirS, nirK, napA, napB, nxrA, nxrB, nrfA, nrfH, nirB, nirD, hdh, nifD, nifH, nifK, amoA, amoB, hao, amoC* |
| Carbon metabolism | *sdhA, acnA, korA/oorA/oforA, korC/oorC, korB/oorB/oforB, korD/oorD, fumA/fumB, sucD, mdh, fhs, folD, metF, acsE, acsB, rbcL, rbcS, aclA, aclB, porA, porB, porD, ppc, ldhA, pycB, pycA, fbp, ppdK, ppsA, pckA, eno, apgM, fbaB, gpmI, gpmA, pfk/pfp, pgk, gapA, tpiA, glk, fbaA* |
| Both of these two metabolisms | *glnA, ureC, aceA, gdhA, acs* |

**Table S8.** The amount of charge Q (μC) passing through the circuit in each group and the total amount of substance n (μmol) transferred electrons.

| Process | Group D | | Group J | | Group Z | | Group S | |
| --- | --- | --- | --- | --- | --- | --- | --- | --- |
|  | Q（μC） | n（μmol） | Q（μC） | n（μmol） | Q（μC） | n（μmol） | Q（μC） | n（μmol） |
| EAC | 842.6 | 0.0087 | 1236.1 | 0.0128 | 1593.9 | 0.0165 | 1853.0 | 0.0192 |
|  | 3444.8 | 0.0357 | 2620.9 | 0.0272 | 2537.6 | 0.0263 | 2665.9 | 0.0276 |
|  | 4211.8 | 0.0436 | 3752.9 | 0.0389 | 3742.5 | 0.0387 | 3809.0 | 0.0395 |
|  | 6726.3 | 0.0697 | 7011.2 | 0.0727 | 3773.3 | 0.0391 | 4225.2 | 0.0438 |
| EDC | -2276.9 | -0.0236 | -1910.6 | -0.0198 | -1640.7 | -0.0170 | -2100.7 | -0.0218 |
|  | -2253.5 | -0.0234 | -1881.6 | -0.0195 | -3071.4 | -0.0318 | -1974.7 | -0.0205 |
|  | -3684.9 | -0.0382 | -3528.6 | -0.0366 | -3187.7 | -0.0330 | -4523.4 | -0.0469 |
|  | -6531.7 | -0.0677 | -4874.0 | -0.0505 | -4063.6 | -0.0421 | -4382.8 | -0.0454 |

**Table S9.** Supplementary information for metagenomic sequencing**.**

| Indicator | Value |
| --- | --- |
| DNA concentration | 10.28–16.28 ng/μl |
| DNA purity | Qualified by agarose gel electrophoresis |
| PCR cycles | 12–15 cycles |
| Sequencing depth | 13.7 Gb clean data per sample |
| Number of effective reads | 91.1 million clean reads per sample |
| Sequencing quality indicator | Q30 > 85% |

**Table S10.** Pairwise Pearson correlations among environmental variables (lower triangular) and Mantel test results for nitrogen‑related gene modules. Each cell shows the significance based on FDR‑adjusted *q* values, with (raw *p*, FDR *q*) in parentheses.*** denotes *q* < 0.001, ** denotes *q* < 0.01, * *q* < 0.05, ns denotes not significant.

| Variable | T | DO | pH | ORP | EC | TDS | NTU | NH_4_^+^ | COD | TN |
| --- | --- | --- | --- | --- | --- | --- | --- | --- | --- | --- |
| T | — |  |  |  |  |  |  |  |  |  |
| DO | ***(<0.001,<0.001) | — |  |  |  |  |  |  |  |  |
| pH | ns(0.057,0.094) | ns(0.248,0.327) | — |  |  |  |  |  |  |  |
| ORP | *(0.032,0.047) | ns(0.121,0.172) | ns(0.395,0.472) | — |  |  |  |  |  |  |
| EC | ns(0.176,0.235) | ns(0.352,0.423) | ns(0.083,0.124) | ns(0.184,0.245) | — |  |  |  |  |  |
| TDS | ns(0.279,0.348) | ns(0.418,0.497) | ns(0.148,0.212) | ns(0.092,0.134) | ***(<0.001,<0.001) | — |  |  |  |  |
| NTU | **(0.001,0.002) | *(0.014,0.026) | ns(0.331,0.403) | ns(0.223,0.291) | ns(0.037,0.053) | *(0.028,0.044) | — |  |  |  |
| NH_4_^+^ | ns(0.419,0.498) | **(0.006,0.011) | ns(0.104,0.147) | **(0.002,0.004) | ns(0.253,0.327) | ns(0.187,0.254) | ns(0.069,0.101) | — |  |  |
| COD | ns(0.781,0.824) | **(0.004,0.009) | *(0.019,0.034) | ns(0.379,0.456) | ns(0.107,0.148) | ns(0.166,0.229) | ns(0.605,0.673) | ns(0.132,0.183) | — |  |
| TN | ns(0.524,0.598) | ns(0.077,0.114) | ns(0.205,0.273) | ns(0.307,0.382) | ns(0.068,0.099) | ns(0.141,0.203) | ns(0.453,0.527) | ***(<0.001,<0.001) | ns(0.056,0.081) | — |
| Denitrification | ns(0.101,0.141) | ns(0.502,0.573) | ns(0.249,0.328) | ns(0.148,0.208) | ns(0.552,0.624) | ns(0.603,0.672) | ns(0.398,0.475) | ns(0.281,0.354) | ns(0.583,0.652) | ns(0.704,0.761) |
| Nitrification | ***(<0.001,<0.001) | *(0.029,0.044) | *(0.013,0.023) | ns(0.082,0.122) | ns(0.332,0.405) | ns(0.397,0.474) | ns(0.505,0.576) | ns(0.098,0.138) | *(0.011,0.019) | ns(0.061,0.089) |
| Nitrogen fixation | ns(0.321,0.397) | ns(0.277,0.351) | ns(0.451,0.525) | **(0.003,0.007) | ns(0.153,0.213) | ns(0.379,0.458) | ns(0.702,0.763) | ns(0.039,0.057) | *(0.008,0.016) | ns(0.217,0.286) |

**Table S11.** The global attributes of co-occurrence networks.

|  | F3. B | F4. C | F5. C | F6. E |
| --- | --- | --- | --- | --- |
| Number of nodes | 39 | 16 | 65 | 76 |
| Number of edges | 339 | 27 | 602 | 568 |
| Average degree | 17.38 | 2.57 | 18.49 | 14.95 |
| Average weighted degree | 14.97 | 2.05 | 15.45 | 12.39 |
| Graph density | 0.46 | 0.13 | 0.29 | 0.20 |
| Modularity index | 0.09 | 0.37 | 0.16 | 0.26 |
| Average clustering coefficient | 0.79 | 0.53 | 0.70 | 0.67 |
| Average path length | 1.71 | 2.26 | 2.15 | 2.62 |

**Table S12.** Microbial functional gene annotation.

| Gene | Function | KO ID |
| --- | --- | --- |
| *nosZ* | Nitrous oxide reductase | K00376 |
| *norB* | Nitric oxide reductase | K04561 |
| *norC* | Nitric oxide reductase | K02305 |
| *nirS* | Cytochrome cd1-type nitrite reductase | K15864 |
| *nirK* | Copper-containing nitrite reductase | K00368 |
| *napA* | Periplasmic nitrate reductase catalytic subunit | K02567 |
| *napB* | Periplasmic nitrate reductase cytochrome c subunit | K02568 |
| *nxrA* | Nitrite oxidoreductase alpha subunit | K00370 |
| *nxrB* | Nitrite oxidoreductase beta subunit | K00371 |
| *nrfA* | Cytochrome c nitrite reductase catalytic subunit | K03385 |
| *nrfH* | Cytochrome c nitrite reductase subunit | K15876 |
| *nirB* | NADH-dependent nitrite reductase large subunit | K00362 |
| *nirD* | NADH-dependent nitrite reductase small subunit | K00363 |
| *hdh* | Hydrazine dehydrogenase | K20935 |
| *nifD* | Nitrogenase molybdenum-iron protein alpha chain | K02586 |
| *nifH* | Nitrogenase iron protein | K02588 |
| *nifK* | Nitrogenase molybdenum-iron protein beta chain | K02591 |
| *amoA* | Ammonia monooxygenase alpha subunit | K10944 |
| *amoB* | Ammonia monooxygenase beta subunit | K10945 |
| *hao* | Hydroxylamine dehydrogenase | K10535 |
| *amoC* | Ammonia monooxygenase gamma subunit | K10946 |
| *gdhA* | Glutamate dehydrogenase | K00262 |
| *glnA* | Glutamine synthetase | K01915 |
| *ureC* | Urease alpha subunit | K01428 |
| *sdhA* | Succinate dehydrogenase flavoprotein subunit | K00239 |
| *acnA* | Aconitate hydratase | K01681 |
| *fumA* | Fumarate hydratase class I | K01676 |
| *fumB* | Fumarate hydratase class I | K01676 |
| *sucD* | Succinyl-CoA synthetase alpha subunit | K01902 |
| *mdh* | Malate dehydrogenase | K00024 |
| *korA/oorA/oforA* | 2-oxoglutarate/2-oxoacid ferredoxin oxidoreductase alpha subunit | K00174 |
| *korC/oorC* | 2-oxoglutarate/2-oxoacid ferredoxin oxidoreductase gamma subunit | K00177 |
| *korB/oorB/oforB* | 2-oxoglutarate/2-oxoacid ferredoxin oxidoreductase beta subunit | K00175 |
| *korD/oorD* | 2-oxoglutarate/2-oxoacid ferredoxin oxidoreductase delta subunit | K00176 |
| *ppc* | Phosphoenolpyruvate carboxylase | K01595 |
| *ldhA* | D-lactate dehydrogenase | K03778 |
| *pycB* | Pyruvate carboxylase beta subunit | K01960 |
| *pycA* | Pyruvate carboxylase alpha subunit | K01959 |
| *porA* | Pyruvate ferredoxin oxidoreductase alpha subunit | K00169 |
| *porB* | Pyruvate ferredoxin oxidoreductase beta subunit | K00170 |
| *porD* | Pyruvate ferredoxin oxidoreductase delta subunit | K00172 |
| *fbp* | Fructose-1,6-bisphosphatase | K03841 |
| *ppdK* | Pyruvate phosphate dikinase | K01006 |
| *ppsA* | Phosphoenolpyruvate synthase | K01007 |
| *pckA* | Phosphoenolpyruvate carboxykinase (ATP) | K01610 |
| *eno* | Enolase | K01689 |
| *apgM* | Cofactor-independent phosphoglycerate mutase | K15635 |
| *fbaB* | Fructose-bisphosphate aldolase class I | K11645 |
| *gpmI* | Cofactor-independent phosphoglycerate mutase | K15633 |
| *gpmA* | Cofactor-dependent phosphoglycerate mutase | K01834 |
| *pfk/pfp* | 6-phosphofructokinase / pyrophosphate-dependent phosphofructokinase | K00850 / K00895 |
| *pgk* | Phosphoglycerate kinase | K00927 |
| *gapA* | Glyceraldehyde-3-phosphate dehydrogenase (GAPDH) | K00134 |
| *tpiA* | Triosephosphate isomerase | K01803 |
| *glk* | Glucokinase | K00845 |
| *fbaA* | Fructose-bisphosphate aldolase class II | K01624 |
| *aceA* | Isocitrate lyase | K01637 |
| *acs* | Acetyl-CoA synthetase | K01895 |
| *acsE* | Acetyl-CoA synthetase | K01895 |
| *acsB* | Acetyl-CoA synthetase / other acyl-CoA ligase | K01895 / K20034 |
| *rbcL* | Ribulose-1,5-bisphosphate carboxylase/oxygenase large subunit | K01601 |
| *rbcS* | Ribulose-1,5-bisphosphate carboxylase/oxygenase small subunit | K01602 |
| *aclA* | ATP-citrate lyase alpha subunit | K15230 |
| *aclB* | ATP-citrate lyase beta subunit | K15231 |
| *fhs* | Formate-tetrahydrofolate ligase | K01938 |
| *folD* | Methylenetetrahydrofolate dehydrogenase/cyclohydrolase | K01491 |
| *metF* | Methylenetetrahydrofolate reductase (MTHFR) | K00297 |
| *afuA* | Iron(III) ABC transport system substrate-binding protein | K02012 |
| *afuB* | Iron(III) ABC transport system permease protein | K02011 |
| *afuC* | Iron(III) ABC transport system ATP-binding protein | K02010 |
| *feoA* | Ferrous iron transporter A | K04758 |
| *feoB* | Ferrous iron transporter B | K04759 |
| *sitA* | Manganese/iron ABC transport system substrate-binding protein | K11604 |
| *sitB* | Manganese/iron ABC transport system ATP-binding protein | K11607 |
| *sitC* | Manganese/iron ABC transport system permease protein | K11605 |
| *mbfA* | Membrane-bound ferritin | K22737 |
| *efeU* | High-affinity iron transporter | K07243 |
| *frdB* | Fumarate reductase iron-sulfur protein subunit | K00245 |
| *erpA* | Iron-sulfur cluster insertion protein | K15724 |
| *iscA* | Iron-sulfur cluster assembly protein | K13628 |
| *iscR* | Iron-sulfur cluster assembly transcription factor | K13643 |
| *petA* | Ubiquinol-cytochrome c reductase iron-sulfur subunit | K00411 |
| *petB* | Ubiquinol-cytochrome c reductase cytochrome b subunit | K00412 |

**Figure S1**. (A) Sampling tools. (B) The sediment sample. (C) and (D) The process of sampling.

**Figure S2**. Line graphs of water quality parameters.

**Figure S3**. Circos plot of the dominant microbial phyla across sampling sites.

**Figure S4**. Circos plot of main KEGG pathways across sampling sites.

**Figure S5**. Analysis statistics of KEGG pathways.

**Figure S6**. Summary of the role of iron-respiring bacteria in sediment carbon and nitrogen cycles.

**Figure S7**. The sampling analysis flowchart.

**Figure S8**. The VPA of effects of environmental factors on the carbon cycles.

**Figure S9**.The correlation between *sitA* and the cycle genes.
